# Supplementary material for: Small Molecular Drug Screening Based on Clinical Therapeutic Effect
Source: Molecules. 2022 Jul 27;27(15):4807. doi: 10.3390/molecules27154807 (PMC9369618; doi:10.3390/molecules27154807)
Supplement: Supplementary file 1 [file molecules-27-04807-s001.zip › molecules-1811241-supplementary.pdf]

molecules MDPI

Article

Small Molecular Drug Screening Based on Clinical Therapeutic Effect

Cai Zhong, Jiali Ai, Yaxin Yang, Fangyuan Ma, Wei Sun *

Supplementary Table S1.

Table S1. Molecules information included in four molecular sets.

No.	Name	Category	No.	Name	Category
1	Aceclofenac[1234]	Analgesic	511	Ceftaroline fosamil[1234]	Antibacterial
2	Acemetacin[1234]	Analgesic	512	Ceftazidime[1234]	Antibacterial
3	Acetaminophen[1234]	Analgesic	513	Cefteram[1234]	Antibacterial
4	Acetanilide[1234]	Analgesic	514	Ceftezole[1234]	Antibacterial
5	Acetyldihydrocodeine[1234]	Analgesic	515	Ceftibuten[1234]	Antibacterial
6	Alclofenac[1234]	Analgesic	516	Ceftiofur[123]	Antibacterial
7	Alfentanil[1234]	Analgesic	517	Ceftiolene[1234]	Antibacterial
8	Allobarbital[12]	Analgesic	518	Ceftizoxime[1234]	Antibacterial
9	Alminoprofen[1234]	Analgesic	519	Ceftobiprole[1234]	Antibacterial
10	Almotriptan[1234]	Analgesic	520	Unii-yxv28V1B07[1234]	Antibacterial
11	Alphaprodine[1234]	Analgesic	521	Ceftolozane[1234]	Antibacterial
12	Aminophenazone[1234]	Analgesic	522	Ceftriaxone[1234]	Antibacterial
13	Amitriptyline[1234]	Analgesic	523	Cefuroxime[1234]	Antibacterial
14	Ampiroxicam[1234]	Analgesic	524	Ceftin[1234]	Antibacterial
15	Ampyrone[1234]	Analgesic	525	Cefuzonam[1234]	Antibacterial
16	Anileridine[1234]	Analgesic	526	Cephalosporin C[1234]	Antibacterial
17	Antipyrine[1234]	Analgesic	527	Cethromycin[1234]	Antibacterial
18	Articaine[12]	Analgesic	528	Chloramphenicol[1234]	Antibacterial
19	Aspirin[1234]	Analgesic	529	Chlortetracycline[1234]	Antibacterial
20	Azapropazone[1234]	Analgesic	530	Cinoxacin[1234]	Antibacterial
21	Bendazac[1234]	Analgesic	531	Ciprofloxacin[1234]	Antibacterial
22	Benorilate[1234]	Analgesic	532	Clarithromycin[1234]	Antibacterial
23	Benoxaprofen[1234]	Analgesic	533	Clavulanic acid[1234]	Antibacterial
24	Benzocaine[1]	Analgesic	534	Clinafloxacin[1234]	Antibacterial
25	Benzydamine[1234]	Analgesic	535	Clindamycin[1234]	Antibacterial
26	Benzylmorphine[1234]	Analgesic	536	Clofoctol[1234]	Antibacterial
27	Bezitramide[1234]	Analgesic	537	Clometocillin[1234]	Antibacterial
28	Bromfenac[1234]	Analgesic	538	Clomocycline[1234]	Antibacterial
29	Bucetin[1234]	Analgesic	539	Cloxacillin[1234]	Antibacterial
30	Bufexamac[1234]	Analgesic	540	Colistin[1234]	Antibacterial
31	Bumadizone[1234]	Analgesic	541	Cycloserine[1234]	Antibacterial
32	Buprenorphine[1234]	Analgesic	542	Dalfopristin[123]	Antibacterial
33	Butanilicaine[1]	Analgesic	543	Danofloxacin[123]	Antibacterial
34	Butorphanol[1234]	Analgesic	544	Dapsone[12]	Antibacterial
35	Camphor[1234]	Analgesic	545	Daptomycin[1234]	Antibacterial
36	Cannabidiol[1234]	Analgesic	546	Deacetoxycephalosporin C[123]	Antibacterial
37	Capsaicin[123]	Analgesic	547	Delafloxacin[1234]	Antibacterial
38	Carbamazepine	Analgesic	548	Demeclocycline[1234]	Antibacterial
39	Carfentanil[123]	Analgesic	549	Dibekacin[1234]	Antibacterial
40	Carisoprodol[1]	Analgesic	550	Dicloxacillin[1234]	Antibacterial
41	Chloroprocaine[12]	Analgesic	551	Difloxacin[123]	Antibacterial
42	Chlorzoxazone[1]	Analgesic	552	Dihydrostreptomycin[1234]	Antibacterial

43	Cinchocaine[12]	Analgesic	553	Dirithromycin[1234]	Antibacterial
44	Clonidine[1234]	Analgesic	554	Doripenem[1234]	Antibacterial
45	Cocaine[1]	Analgesic	555	Doxycycline[1234]	Antibacterial
46	Codeine[1234]	Analgesic	556	Enoxacin[1234]	Antibacterial
47	Cyclobenzaprine[1]	Analgesic	557	Enrofloxacin[12]	Antibacterial
48	Cyclomethycaine[1]	Analgesic	558	Eperezolid[123]	Antibacterial
49	Desomorphine[1234]	Analgesic	559	Epicillin[1234]	Antibacterial
50	Dexmedetomidine[12]	Analgesic	560	Eravacycline[1234]	Antibacterial
51	Dextromoramide[1234]	Analgesic	561	Ertapenem[1234]	Antibacterial
52	Dextropropoxyphene[1234]	Analgesic	562	Erythromycin[1234]	Antibacterial
53	Dezocine[1234]	Analgesic	563	Faropenem[1234]	Antibacterial
54	Diacerein[1234]	Analgesic	564	Finafloxacin[1234]	Antibacterial
55	Diamorphine[1234]	Analgesic	565	Fleroxacin[1234]	Antibacterial
56	Diclofenac[1234]	Analgesic	566	Flomoxef[1234]	Antibacterial
57	Diflunisal[1234]	Analgesic	567	Florfenicol[123]	Antibacterial
58	Dihydrocodeine[1234]	Analgesic	568	Flucloxacillin[1234]	Antibacterial
59	Dihydroergotamine[1234]	Analgesic	569	Flumequine[1234]	Antibacterial
60	Dihydromorphine[1234]	Analgesic	570	Flurithromycin[1234]	Antibacterial
61	Dimenoxadol[1234]	Analgesic	571	Fosfomycin[1234]	Antibacterial
62	Dimethocaine[1]	Analgesic	572	Framycetin[1234]	Antibacterial
63	Dimetotiazine[1234]	Analgesic	573	Furazidin[1234]	Antibacterial
64	Dipipanone[1234]	Analgesic	574	Furazolidone[123]	Antibacterial
65	Dipyrocetyl[1234]	Analgesic	575	Fusidic acid[1234]	Antibacterial
66	Dronabinol[1234]	Analgesic	576	Garenoxacin[12]	Antibacterial
67	Droxicam[1234]	Analgesic	577	Gatifloxacin[1234]	Antibacterial
68	DSP-2230[12]	Analgesic	578	Gemifloxacin[1234]	Antibacterial
69	Dyclonine[1]	Analgesic	579	Gentamicin[1234]	Antibacterial
70	Eletriptan[1234]	Analgesic	580	Grepafloxacin[1234]	Antibacterial
71	Ergotamine[1234]	Analgesic	581	Hetacillin[1234]	Antibacterial
72	Ethenzamide[1234]	Analgesic	582	Hygromycin B[123]	Antibacterial
73	Ethoheptazine[1234]	Analgesic	583	Ibafloxacin[1234]	Antibacterial
74	Ethylmorphine[1234]	Analgesic	584	Iclaprim[1234]	Antibacterial
75	Etidocaine[1]	Analgesic	585	Imipenem[1234]	Antibacterial
76	Etodolac[1234]	Analgesic	586	Isepamicin[1234]	Antibacterial
77	Etofenamate[1234]	Analgesic	587	Isoniazid[1234]	Antibacterial
78	Etoricoxib[1234]	Analgesic	588	Josamycin[1234]	Antibacterial
79	Eugenol[12]	Analgesic	589	Kanamycin[1234]	Antibacterial
80	Fenbufen[1234]	Analgesic	590	Latamoxef[1234]	Antibacterial
81	Fenoprofen[1234]	Analgesic	591	Lefamulin[1234]	Antibacterial
82	Fentanyl[12]	Analgesic	592	Lincomycin[1234]	Antibacterial
83	Fentiazac[1234]	Analgesic	593	Linezolid[1234]	Antibacterial
84	Feprazone[1234]	Analgesic	594	Lomefloxacin[1234]	Antibacterial
85	Floctafenine[1234]	Analgesic	595	Loracarbef[1234]	Antibacterial
86	Flufenamic acid[1234]	Analgesic	596	Lymecycline[1234]	Antibacterial
87	Flumedroxone[1234]	Analgesic	597	Mandelic acid[1234]	Antibacterial
88	Flunoxaprofen[1234]	Analgesic	598	Marbofloxacin[123]	Antibacterial
89	Flupirtine[1234]	Analgesic	599	Meclocycline[1234]	Antibacterial
90	Fluproquazone[123]	Analgesic	600	Meropenem[1234]	Antibacterial
91	Flurbiprofen[1234]	Analgesic	601	Metacycline[1234]	Antibacterial
92	Frovatriptan[1234]	Analgesic	602	Metampicillin[1234]	Antibacterial
93	Funapide[12]	Analgesic	603	Methenamine[123]	Antibacterial
94	Gabapentin[1234]	Analgesic	604	Methicillin[1234]	Antibacterial
95	Gabapentin enacarbil[1234]	Analgesic	605	Metronidazole[1234]	Antibacterial
96	Glafenine[1234]	Analgesic	606	Mezlocillin[1234]	Antibacterial
97	Guacetisal[1234]	Analgesic	607	Midecamycin[1234]	Antibacterial
98	Halothane[1]	Analgesic	608	Minocycline[1234]	Antibacterial
99	Hydrocodone[1234]	Analgesic	609	Miocamycin[1234]	Antibacterial

100	Hydromorphinol[1234]	Analgesic	610	Moxifloxacin[1234]	Antibacterial
101	Hydromorphone[1234]	Analgesic	611	Mupirocin[1234]	Antibacterial
102	Ibuproxam[1234]	Analgesic	612	Nadifloxacin[1234]	Antibacterial
103	Indometacin[1234]	Analgesic	613	Nafcillin[1234]	Antibacterial
104	Indomethacin farnesil[123]	Analgesic	614	Nalidixic acid[1234]	Antibacterial
105	Indoprofen[1234]	Analgesic	615	Nemonoxacin[1234]	Antibacterial
106	Iprazochrome[1234]	Analgesic	616	Netilmicin[1234]	Antibacterial
107	Isoflurane[12]	Analgesic	617	Nifurtoinol[1234]	Antibacterial
108	Kebuzone[1234]	Analgesic	618	Nitrofurantoin[1234]	Antibacterial
109	Ketamine[12]	Analgesic	619	Nitroxoline[1234]	Antibacterial
110	Ketobemidone[1234]	Analgesic	620	Nocardicin A[1234]	Antibacterial
111	Ketorolac[1234]	Analgesic	621	Norfloxacin[1234]	Antibacterial
112	Lacosamide[1]	Analgesic	622	Nourseothricin[12]	Antibacterial
113	Levorphanol[1234]	Analgesic	623	Novobiocin[1234]	Antibacterial
114	Lisuride[12]	Analgesic	624	Oleandomycin[123]	Antibacterial
115	Lofentanil[1234]	Analgesic	625	Omadacycline[1234]	Antibacterial
116	Lonazolac[1234]	Analgesic	626	Orbifloxacin[123]	Antibacterial
117	Lornoxicam[1234]	Analgesic	627	Oritavancin[1234]	Antibacterial
118	Loxoprofen[1234]	Analgesic	628	Ornidazole[1234]	Antibacterial
119	Lumiracoxib[1234]	Analgesic	629	Oxacillin[1234]	Antibacterial
120	Meclofenamic acid[1234]	Analgesic	630	Oxolinic acid[12]	Antibacterial
121	Mefenamic acid[1234]	Analgesic	631	Oxytetracycline[1234]	Antibacterial
122	Meloxicam[1234]	Analgesic	632	Ozenoxacin[1234]	Antibacterial
123	Menthol[123]	Analgesic	633	Panipenem[1234]	Antibacterial
124	Meperidine[1234]	Analgesic	634	Paromomycin[1234]	Antibacterial
125	Mephenoxalone[12]	Analgesic	635	Pazufloxacin[1234]	Antibacterial
126	Mepivacaine[1]	Analgesic	636	Pefloxacin[1234]	Antibacterial
127	Meptazinol[1234]	Analgesic	637	Penamecillin[1234]	Antibacterial
128	Metabutethamine[1]	Analgesic	638	Pheneticillin[1234]	Antibacterial
129	Metamizole[1234]	Analgesic	639	Phenoxymethylpenicillin[1234]	Antibacterial
130	Metethoheptazine[1234]	Analgesic	640	Pipemidic acid[12]	Antibacterial
131	Methadone[1234]	Analgesic	641	Piperacillin[1234]	Antibacterial
132	Metheptazine[1234]	Analgesic	642	Pirlimycin[123]	Antibacterial
133	Methocarbamol[123]	Analgesic	643	Piromidic acid[1234]	Antibacterial
134	Methoxyflurane[123]	Analgesic	644	Pivampicillin[1234]	Antibacterial
135	Methyl salicylate[1234]	Analgesic	645	Pivmecillinam[1234]	Antibacterial
136	Methysergide[123]	Analgesic	646	Plazomicin[1234]	Antibacterial
137	Metopon[1234]	Analgesic	647	Polymyxin B[1234]	Antibacterial
138	Mirogabalin[1234]	Analgesic	648	Posizolid[1234]	Antibacterial
139	Miroprofen[1234]	Analgesic	649	Pradofloxacin[123]	Antibacterial
140	Mofebutazone[1234]	Analgesic	650	Prontosil[1234]	Antibacterial
141	Morniflumate[1234]	Analgesic	651	Propicillin[1234]	Antibacterial
142	Morphine[1234]	Analgesic	652	Prulifloxacin[1234]	Antibacterial
143	Morpholine[12]	Analgesic	653	Puromycin[12]	Antibacterial
144	Nabilone[12]	Analgesic	654	Quinupristin[123]	Antibacterial
145	Nabumetone[1234]	Analgesic	655	Radezolid[1234]	Antibacterial
146	Nalbuphine[1234]	Analgesic	656	Ranbezolid[123]	Antibacterial
147	Naproxcinod[1234]	Analgesic	657	Relebactam[1234]	Antibacterial
148	Naproxen[1234]	Analgesic	658	Retapamulin[1234]	Antibacterial
149	Naratriptan[1234]	Analgesic	659	Ribostamycin[1234]	Antibacterial
150	Nefopam[1234]	Analgesic	660	Rifabutin[1234]	Antibacterial
151	NFEPP[1234]	Analgesic	661	Rifalazil[1234]	Antibacterial
152	Nicocodeine[1234]	Analgesic	662	Rifamycin[1234]	Antibacterial
153	Nicodicodine[1234]	Analgesic	663	Rifapentine[1234]	Antibacterial
154	Nicomorphine[1234]	Analgesic	664	Rifaximin[1234]	Antibacterial
155	Nifenazone[1234]	Analgesic	665	Ritipenem[1234]	Antibacterial
156	Niflumic acid[1234]	Analgesic	666	Rokitamycin[1234]	Antibacterial

157	Nimesulide[1234]	Analgesic	667	Rolitetracycline[1234]	Antibacterial
158	Nitrous Oxide[12]	Analgesic	668	Rosoxacin[1234]	Antibacterial
159	Norpipanone[1234]	Analgesic	669	Roxithromycin[1234]	Antibacterial
160	Nortilidine[1234]	Analgesic	670	Rufloxacin[1234]	Antibacterial
161	Orphenadrine[123]	Analgesic	671	Sarafloxacin[123]	Antibacterial
162	Oxametacin[123]	Analgesic	672	Sarecycline[1234]	Antibacterial
163	Oxaprozin[1234]	Analgesic	673	Sisomicin[1234]	Antibacterial
164	Oxetorone[1234]	Analgesic	674	Sitafloxacin[1234]	Antibacterial
165	Oxycodone[1234]	Analgesic	675	Solithromycin[1234]	Antibacterial
166	Oxymorphone[1234]	Analgesic	676	Sparfloxacin[1234]	Antibacterial
167	Oxyphenbutazone[1234]	Analgesic	677	Spectinomycin[1234]	Antibacterial
168	Parecoxib[1234]	Analgesic	678	Spiramycin[1234]	Antibacterial
169	Pentazocine[123]	Analgesic	679	Streptomycin[1234]	Antibacterial
170	PF-05089771[1234]	Analgesic	680	Sulbactam[1234]	Antibacterial
171	Phenacetin[1234]	Analgesic	681	Sulbenicillin[1234]	Antibacterial
172	Phenadoxone[1234]	Analgesic	682	Sulfacetamide[1234]	Antibacterial
173	Phenazocine[1234]	Analgesic	683	Sulfadiazine[1234]	Antibacterial
174	Phenazopyridine[123]	Analgesic	684	Sulfadicramide[123]	Antibacterial
175	Phenol[12]	Analgesic	685	Sulfadimethoxine[1234]	Antibacterial
176	Phenoperidine[1234]	Analgesic	686	Sulfadimidine[1234]	Antibacterial
177	Phenylbutazone[1234]	Analgesic	687	Sulfadoxine[1234]	Antibacterial
178	Piminodine[1234]	Analgesic	688	Sulfafurazole[1234]	Antibacterial
179	Piperocaine[1]	Analgesic	689	Sulfaisodimidine[1234]	Antibacterial
180	Piritramide[1234]	Analgesic	690	Sulfalene[1234]	Antibacterial
181	Piroxicam[1234]	Analgesic	691	Sulfamazone[12]	Antibacterial
182	Pirprofen[1234]	Analgesic	692	Sulfamerazine[1234]	Antibacterial
183	Pizotifen[123]	Analgesic	693	Sulfameter[1234]	Antibacterial
184	Pregabalin[123]	Analgesic	694	Sulfamethizole[1234]	Antibacterial
185	Prilocaine[12]	Analgesic	695	Sulfamethoxazole[1234]	Antibacterial
186	Procaine[12]	Analgesic	696	Sulfamethoxypyridazine[1234]	Antibacterial
187	Proglumetacin[1234]	Analgesic	697	Sulfametomidine[1234]	Antibacterial
188	Proglumide[123]	Analgesic	698	Sulfametrole[1234]	Antibacterial
189	Proheptazine[1234]	Analgesic	699	Sulfamoxole[1234]	Antibacterial
190	Propacetamol[1234]	Analgesic	700	Sulfanilamide[1234]	Antibacterial
191	Proparacaine[1]	Analgesic	701	Sulfanitran[123]	Antibacterial
192	Propiram[1234]	Analgesic	702	Sulfaperin[1234]	Antibacterial
193	Propoxycaine[1]	Analgesic	703	Sulfaphenazole[1234]	Antibacterial
194	Propyphenazone[1234]	Analgesic	704	Sulfapyridine[1234]	Antibacterial
195	Proquazone[1234]	Analgesic	705	Sulfathiazole[1234]	Antibacterial
196	Proxibarbal[123]	Analgesic	706	Sulfathiourea[1234]	Antibacterial
197	Remifentanil[1]	Analgesic	707	Sultamicillin[1234]	Antibacterial
198	Rimazolium[1234]	Analgesic	708	Sutezolid[1234]	Antibacterial
199	Rizatriptan[123]	Analgesic	709	Talampicillin[1234]	Antibacterial
200	Rofecoxib[1234]	Analgesic	710	Tazobactam[1234]	Antibacterial
201	Ropivacaine[123]	Analgesic	711	Tebipenem pivoxil[1234]	Antibacterial
202	Salicin[1234]	Analgesic	712	Tedizolid[1234]	Antibacterial
203	Salicylamide[1234]	Analgesic	713	Telavancin[1234]	Antibacterial
204	Salsalate[1234]	Analgesic	714	Telithromycin[1234]	Antibacterial
205	Saxitoxin[1]	Analgesic	715	Temafloxacin[1234]	Antibacterial
206	Sevoflurane[12]	Analgesic	716	Temocillin[1234]	Antibacterial
207	Spilanthol[12]	Analgesic	717	Tetracycline[1234]	Antibacterial
208	Sufentanil[12]	Analgesic	718	Tetroxoprim[1234]	Antibacterial
209	Sulindac[1234]	Analgesic	719	Thiamphenicol[1234]	Antibacterial
210	Sumatriptan[1234]	Analgesic	720	Tiamulin[123]	Antibacterial
211	Suprofen[1234]	Analgesic	721	Ticarcillin[1234]	Antibacterial
212	Tapentadol[1234]	Analgesic	722	Tigecycline[1234]	Antibacterial
213	Tenoxicam[1234]	Analgesic	723	Tigemonam[1234]	Antibacterial

214	Tetracaine[12]	Analgesic	724	Tinidazole[1234]	Antibacterial
215	Tetrodotoxin[123]	Analgesic	725	Tobramycin[1234]	Antibacterial
216	Thebacon[1234]	Analgesic	726	Tosufloxacin[1234]	Antibacterial
217	Thiopental[12]	Analgesic	727	Trimethoprim[1234]	Antibacterial
218	Tiaprofenic acid[1234]	Analgesic	728	Troleandomycin[1234]	Antibacterial
219	Tilidine[1234]	Analgesic	729	Trovafloxacin[1234]	Antibacterial
220	Tolfenamic acid[1234]	Analgesic	730	Tyrothricin[1234]	Antibacterial
221	Tolmetin[1234]	Analgesic	731	Vaborbactam[1234]	Antibacterial
222	Tramadol[1234]	Analgesic	732	Valnemulin[1234]	Antibacterial
223	Trichloroethylene[12]	Analgesic	733	Vancomycin[1234]	Antibacterial
224	Trimecaine[12]	Analgesic	734	Verdamicin[1234]	Antibacterial
225	Valdecoxib[1234]	Analgesic	735	Xibornol[123]	Antibacterial
226	Viminol[1234]	Analgesic	736	Abacavir[1234]	Antiviral
227	Zolmitriptan[1234]	Analgesic	737	Aciclovir[1234]	Antiviral
228	Zomepirac[1234]	Analgesic	738	Adefovir[12]	Antiviral
229	Abemaciclib[1234]	Antineoplastic	739	Adefovir dipivoxil[1234]	Antiviral
230	Acalabrutinib[1234]	Antineoplastic	740	Amdoxovir[1234]	Antiviral
231	Aclarubicin[12]	Antineoplastic	741	Amenamevir[1234]	Antiviral
232	Afatinib[1234]	Antineoplastic	742	Amprenavir[1234]	Antiviral
233	Alectinib[1234]	Antineoplastic	743	Apricitabine[1234]	Antiviral
234	Alpelisib[1234]	Antineoplastic	744	Asunaprevir[1234]	Antiviral
235	Altretamine[1234]	Antineoplastic	745	Atazanavir[1234]	Antiviral
236	Alvocidib[123]	Antineoplastic	746	Baloxavir marboxil[1234]	Antiviral
237	Aminolevulinic acid[1234]	Antineoplastic	747	Bevirimat[1234]	Antiviral
238	Aminopropionitrile[1234]	Antineoplastic	748	BI 224436[1234]	Antiviral
239	Aminopterin[1234]	Antineoplastic	749	Bictegravir[1234]	Antiviral
240	Amrubicin[12]	Antineoplastic	750	BIT225[1234]	Antiviral
241	Amsacrine[1234]	Antineoplastic	751	BMS-955176[1234]	Antiviral
242	Anagrelide[1234]	Antineoplastic	752	Boceprevir[1234]	Antiviral
243	Asparagine[1]	Antineoplastic	753	Brivudine[1234]	Antiviral
244	Atrasentan[12]	Antineoplastic	754	Cabotegravir[1234]	Antiviral
245	Axitinib[1234]	Antineoplastic	755	Calanolide A[1234]	Antiviral
246	Azacitidine[1234]	Antineoplastic	756	Cenicriviroc[12]	Antiviral
247	Baricitinib[1234]	Antineoplastic	757	Censavudine[1234]	Antiviral
248	Belinostat[1234]	Antineoplastic	758	Cidofovir[1234]	Antiviral
249	Belotecan[1234]	Antineoplastic	759	Clevudine[1234]	Antiviral
250	Bendamustine[1234]	Antineoplastic	760	Daclatasvir[1234]	Antiviral
251	Bexarotene[1234]	Antineoplastic	761	Darunavir[1234]	Antiviral
252	Binimetinib[1234]	Antineoplastic	762	Dasabuvir[1234]	Antiviral
253	Bleomycin[12]	Antineoplastic	763	Delavirdine[1234]	Antiviral
254	Bortezomib[1234]	Antineoplastic	764	Didanosine[1234]	Antiviral
255	Bosutinib[1234]	Antineoplastic	765	Docosanol[1234]	Antiviral
256	Brigatinib[1234]	Antineoplastic	766	Dolutegravir[1234]	Antiviral
257	Busulfan[1234]	Antineoplastic	767	Doravirine[1234]	Antiviral
258	Cabazitaxel[1234]	Antineoplastic	768	Edoxudine[1234]	Antiviral
259	Cabozantinib[1234]	Antineoplastic	769	Efavirenz[1234]	Antiviral
260	Camptothecin[1234]	Antineoplastic	770	Elbasvir[1234]	Antiviral
261	Capecitabine[1234]	Antineoplastic	771	Elvitegravir[1234]	Antiviral
262	Carboquone[123]	Antineoplastic	772	Elvucitabine[1234]	Antiviral
263	Carfilzomib[1234]	Antineoplastic	773	Emtricitabine[1234]	Antiviral
264	Carmofur[1234]	Antineoplastic	774	Entecavir[1234]	Antiviral
265	Carmustine[1234]	Antineoplastic	775	Epigallocatechin gallate[123]	Antiviral
266	Cediranib[1234]	Antineoplastic	776	Etravirine[1234]	Antiviral
267	Ceritinib[1234]	Antineoplastic	777	Faldaprevir[1234]	Antiviral
268	Chlorambucil[1234]	Antineoplastic	778	Famciclovir[1234]	Antiviral
269	Chlormethine[1234]	Antineoplastic	779	Favipiravir[1234]	Antiviral
270	Cladribine[1234]	Antineoplastic	780	Filociclovir[1234]	Antiviral

271	Clofarabine[1234]	Antineoplastic	781	Fosamprenavir[1234]	Antiviral
272	Cobimetinib[1234]	Antineoplastic	782	Foscarnet[1234]	Antiviral
273	Copanlisib[1234]	Antineoplastic	783	Fosdevirine[1234]	Antiviral
274	Cositecan[1234]	Antineoplastic	784	Fostemsavir[1234]	Antiviral
275	Crizotinib[1234]	Antineoplastic	785	Ganciclovir[1234]	Antiviral
276	Cyclophosphamide[1234]	Antineoplastic	786	Grazoprevir[1234]	Antiviral
277	Dabrafenib[1234]	Antineoplastic	787	Idoxuridine[1234]	Antiviral
278	Dacarbazine[1234]	Antineoplastic	788	Imiquimod[1234]	Antiviral
279	Dacomitinib[1234]	Antineoplastic	789	Indinavir[1234]	Antiviral
280	Dactinomycin[12]	Antineoplastic	790	Islatravir[1234]	Antiviral
281	Dasatinib[1234]	Antineoplastic	791	KP-1461[1234]	Antiviral
282	Daunorubicin[123]	Antineoplastic	792	Lamivudine[1234]	Antiviral
283	Decitabine[1234]	Antineoplastic	793	Laninamivir[1234]	Antiviral
284	Demecolcine[12]	Antineoplastic	794	Letermovir[1234]	Antiviral
285	Docetaxel[1234]	Antineoplastic	795	Lobucavir[1234]	Antiviral
286	Doxifluridine[1234]	Antineoplastic	796	Lopinavir[1234]	Antiviral
287	Elesclomol[1234]	Antineoplastic	797	Maraviroc[1234]	Antiviral
288	Elsamitrucin[12]	Antineoplastic	798	Maribavir[1234]	Antiviral
289	Enasidenib[1234]	Antineoplastic	799	Methisazone[1234]	Antiviral
290	Encorafenib[1234]	Antineoplastic	800	Miltefosine[12]	Antiviral
291	Entinostat[1234]	Antineoplastic	801	MK-2048[1234]	Antiviral
292	Entrectinib[1234]	Antineoplastic	802	Moroxydine[1234]	Antiviral
293	Epacadostat[1234]	Antineoplastic	803	Nelfinavir[1234]	Antiviral
294	Erdafitinib[1234]	Antineoplastic	804	Nevirapine[1234]	Antiviral
295	Eribulin[1234]	Antineoplastic	805	Oseltamivir[1234]	Antiviral
296	Erlotinib[1234]	Antineoplastic	806	Penciclovir[1234]	Antiviral
297	Estramustine[1234]	Antineoplastic	807	Ingavirin[1234]	Antiviral
298	Estramustine phosphate[1234]	Antineoplastic	808	Peramivir[1234]	Antiviral
299	Etoglucid[123]	Antineoplastic	809	Phosphonoacetate[1234]	Antiviral
300	Etoposide[1234]	Antineoplastic	810	Pleconaril[1234]	Antiviral
301	Everolimus[1234]	Antineoplastic	811	Podophyllotoxin[12]	Antiviral
302	Exatecan[1234]	Antineoplastic	812	Raltegravir[1234]	Antiviral
303	Fedratinib[1234]	Antineoplastic	813	Remdesivir[1234]	Antiviral
304	Filgotinib[1234]	Antineoplastic	814	Resiquimod[12]	Antiviral
305	Floxuridine[1234]	Antineoplastic	815	Ribavirin[1234]	Antiviral
306	Fludarabine[1234]	Antineoplastic	816	Rilpivirine[1234]	Antiviral
307	Fluorouracil[1234]	Antineoplastic	817	Rimantadine[1234]	Antiviral
308	Fotemustine[1234]	Antineoplastic	818	Ritonavir[1234]	Antiviral
309	Gefitinib[1234]	Antineoplastic	819	Saquinavir[1234]	Antiviral
310	Gemcitabine[1234]	Antineoplastic	820	Simeprevir[1234]	Antiviral
311	Gilteritinib[1234]	Antineoplastic	821	Sofosbuvir[1234]	Antiviral
312	Gimatecan[123]	Antineoplastic	822	Sorivudine[1234]	Antiviral
313	Hydroxyurea[1234]	Antineoplastic	823	Stampidine[123]	Antiviral
314	Ibrutinib[1234]	Antineoplastic	824	Stavudine[1234]	Antiviral
315	Icotinib[1234]	Antineoplastic	825	Taribavirin[1234]	Antiviral
316	Idarubicin[12]	Antineoplastic	826	Tecovirimat[1234]	Antiviral
317	Idelalisib[1234]	Antineoplastic	827	Telaprevir[1234]	Antiviral
318	Ifosfamide[1234]	Antineoplastic	828	Telbivudine[1234]	Antiviral
319	Imatinib[1234]	Antineoplastic	829	Tenofovir[1234]	Antiviral
320	Infigratinib[1234]	Antineoplastic	830	Tenofovir alafenamide[1234]	Antiviral
321	Irinotecan[1234]	Antineoplastic	831	Tenofovir disoproxil[1234]	Antiviral
322	Ivosidenib[1234]	Antineoplastic	832	Tilorone[12]	Antiviral
323	Ixabepilone[12]	Antineoplastic	833	Tipranavir[1234]	Antiviral
324	Ixazomib[1234]	Antineoplastic	834	TMC-310911[1234]	Antiviral
325	Lapatinib[1234]	Antineoplastic	835	Trifluridine[1234]	Antiviral
326	Larotaxel[123]	Antineoplastic	836	Tromantadine[1234]	Antiviral
327	Larotrectinib[1234]	Antineoplastic	837	Umifenovir[1234]	Antiviral

328	Lenvatinib[1234]	Antineoplastic	838	Valaciclovir[1234]	Antiviral
329	Lestaurtinib[1234]	Antineoplastic	839	Valganciclovir[1234]	Antiviral
330	Lomustine[1234]	Antineoplastic	840	Vicriviroc[123]	Antiviral
331	Lonidamine[1234]	Antineoplastic	841	Zalcitabine[1234]	Antiviral
332	Lorlatinib[1234]	Antineoplastic	842	Zanamivir[1234]	Antiviral
333	Losoxantrone[12]	Antineoplastic	843	Zidovudine[1234]	Antiviral
334	Lucanthone[1]	Antineoplastic	844	Abafungin[1234]	Antifungal
335	Lurtotecan[1234]	Antineoplastic	845	Albaconazole[1234]	Antifungal
336	Mannosulfan[123]	Antineoplastic	846	Amorolfine[1234]	Antifungal
337	Masitinib[123]	Antineoplastic	847	Amphotericin B[1234]	Antifungal
338	Masoprocol[1234]	Antineoplastic	848	Anidulafungin[1234]	Antifungal
339	Melphalan[1234]	Antineoplastic	849	Atovaquone[1234]	Antifungal
340	Mercaptopurine[1234]	Antineoplastic	850	Bifonazole[1234]	Antifungal
341	Methotrexate[123]	Antineoplastic	851	Bromochlorosalicylanilide[1234]	Antifungal
342	Midostaurin[1234]	Antineoplastic	852	Butenafine[1234]	Antifungal
343	Mitobronitol[123]	Antineoplastic	853	Butoconazole[1234]	Antifungal
344	Mitoguazone[1234]	Antineoplastic	854	Caspofungin[1234]	Antifungal
345	Mitomycin[12]	Antineoplastic	855	Chlormidazole[1234]	Antifungal
346	Mitotane[1234]	Antineoplastic	856	Chlorophetanol[1234]	Antifungal
347	Mitoxantrone[12]	Antineoplastic	857	Chlorphenesin[12]	Antifungal
348	Momelotinib[123]	Antineoplastic	858	Ciclopirox[1234]	Antifungal
349	Nelarabine[1234]	Antineoplastic	859	Clotrimazole[12]	Antifungal
350	Neratinib[1234]	Antineoplastic	860	Croconazole[123]	Antifungal
351	Nilotinib[1234]	Antineoplastic	861	Dimazole[1234]	Antifungal
352	Nimustine[1234]	Antineoplastic	862	Eberconazole[1234]	Antifungal
353	Nintedanib[1234]	Antineoplastic	863	Econazole[1234]	Antifungal
354	Niraparib[1234]	Antineoplastic	864	Efinaconazole[1234]	Antifungal
355	Olaparib[1234]	Antineoplastic	865	Ethylparaben[1234]	Antifungal
356	Olmutinib[1234]	Antineoplastic	866	Fenticonazole[1234]	Antifungal
357	Omacetaxine[1234]	Antineoplastic	867	Fluconazole[1234]	Antifungal
358	Oprozomib[123]	Antineoplastic	868	Flucytosine[1234]	Antifungal
359	Ortataxel[123]	Antineoplastic	869	Flutrimazole[1234]	Antifungal
360	Osimertinib[1234]	Antineoplastic	870	Fosfluconazole[1234]	Antifungal
361	Paclitaxel[1234]	Antineoplastic	871	Griseofulvin[12]	Antifungal
362	Pacritinib[123]	Antineoplastic	872	Haloprogin[1234]	Antifungal
363	Palbociclib[1234]	Antineoplastic	873	Hexaconazole[123]	Antifungal
364	Panobinostat[1234]	Antineoplastic	874	Isavuconazole[1234]	Antifungal
365	Pazopanib[123]	Antineoplastic	875	Isoconazole[1234]	Antifungal
366	Pemetrexed[1234]	Antineoplastic	876	Itraconazole[1234]	Antifungal
367	Pemigatinib[1234]	Antineoplastic	877	Ketoconazole[1234]	Antifungal
368	Pentostatin[1234]	Antineoplastic	878	Luliconazole[1234]	Antifungal
369	Pipobroman[1234]	Antineoplastic	879	Mepartricin[12]	Antifungal
370	Pirarubicin[12]	Antineoplastic	880	Micafungin[1234]	Antifungal
371	Pixantrone[12]	Antineoplastic	881	Miconazole[1234]	Antifungal
372	Plicamycin[12]	Antineoplastic	882	Naftifine[1234]	Antifungal
373	Plitidepsin[1234]	Antineoplastic	883	Natamycin[12]	Antifungal
374	Ponatinib[1234]	Antineoplastic	884	Neticonazole[1234]	Antifungal
375	Pralatrexate[1234]	Antineoplastic	885	Nystatin[1234]	Antifungal
376	Pralsetinib[1234]	Antineoplastic	886	Omoconazole[1234]	Antifungal
377	Prednimustine[1234]	Antineoplastic	887	Oxiconazole[1234]	Antifungal
378	Procarbazine[1234]	Antineoplastic	888	Pecilocin[12]	Antifungal
379	Quizartinib[1234]	Antineoplastic	889	Pentamidine[1234]	Antifungal
380	Radotinib[1234]	Antineoplastic	890	Posaconazole[1234]	Antifungal
381	Raltitrexed[1234]	Antineoplastic	891	Pyrrolnitrin[123]	Antifungal
382	Ranimustine[1234]	Antineoplastic	892	Ravuconazole[1234]	Antifungal
383	Regorafenib[1234]	Antineoplastic	893	Sertaconazole[1234]	Antifungal
384	Ribociclib[1234]	Antineoplastic	894	Sulbentine[1234]	Antifungal

385	Ridaforolimus[123]	Antineoplastic	895	Sulconazole[1234]	Antifungal
386	Ripretinib[1234]	Antineoplastic	896	Taurolidine[12]	Antifungal
387	Rociletinib[123]	Antineoplastic	897	Tavaborole[1234]	Antifungal
388	Romidepsin[1234]	Antineoplastic	898	Terbinafine[1234]	Antifungal
389	Rubitecan[12]	Antineoplastic	899	Terconazole[1234]	Antifungal
390	Rucaparib[12]	Antineoplastic	900	Thiabendazole[1234]	Antifungal
391	Ruxolitinib[1234]	Antineoplastic	901	Ticlatone[1234]	Antifungal
392	Selinexor[1234]	Antineoplastic	902	Tioconazole[1234]	Antifungal
393	Selpercatinib[1234]	Antineoplastic	903	Tolciclate[1234]	Antifungal
394	Selumetinib[1234]	Antineoplastic	904	Tolnaftate[1234]	Antifungal
395	Semaxanib[123]	Antineoplastic	905	Tribromometacresol[1234]	Antifungal
396	Semustine[1234]	Antineoplastic	906	Undecylenic acid[1234]	Antifungal
397	Silatecan[123]	Antineoplastic	907	Voriconazole[1234]	Antifungal
398	Sonidegib[1234]	Antineoplastic	908	Acarbose[1234]	Antidiabetic
399	Sorafenib[1234]	Antineoplastic	909	Acetohexamide[1234]	Antidiabetic
400	Streptozocin[12]	Antineoplastic	910	Aleglitazar[1234]	Antidiabetic
401	Sunitinib[1234]	Antineoplastic	911	Alogliptin[1234]	Antidiabetic
402	Talazoparib[1234]	Antineoplastic	912	Anagliptin[1234]	Antidiabetic
403	Tegafur[1234]	Antineoplastic	913	Benfluorex[1234]	Antidiabetic
404	Temozolomide[1234]	Antineoplastic	914	Bexagliflozin[1234]	Antidiabetic
405	Temsirolimus[1234]	Antineoplastic	915	Bromocriptine[1234]	Antidiabetic
406	Teniposide[1234]	Antineoplastic	916	Buformin[1234]	Antidiabetic
407	Tepotinib[1234]	Antineoplastic	917	Canagliflozin[1234]	Antidiabetic
408	Tesetaxel[123]	Antineoplastic	918	Carbutamide[1234]	Antidiabetic
409	Testolactone[1234]	Antineoplastic	919	Chlorpropamide[1234]	Antidiabetic
410	Tezacitabine[123]	Antineoplastic	920	Ciglitazone[12]	Antidiabetic
411	Thiotepa[1234]	Antineoplastic	921	Dapagliflozin[1234]	Antidiabetic
412	Tiazofurine[123]	Antineoplastic	922	Darglitazone[12]	Antidiabetic
413	Tioguanine[12]	Antineoplastic	923	Empagliflozin[1234]	Antidiabetic
414	Tipifarnib[1234]	Antineoplastic	924	Englitazone[1234]	Antidiabetic
415	Tivozanib[1234]	Antineoplastic	925	Epalrestat[12]	Antidiabetic
416	Toceranib[123]	Antineoplastic	926	Ertugliflozin[1234]	Antidiabetic
417	Topotecan[1234]	Antineoplastic	927	Evogliptin[1234]	Antidiabetic
418	Trabectedin[1234]	Antineoplastic	928	Fasiglifam[1234]	Antidiabetic
419	Trametinib[1234]	Antineoplastic	929	Fidarestat[123]	Antidiabetic
420	Treosulfan[1234]	Antineoplastic	930	Gemigliptin[12]	Antidiabetic
421	Triapine[1234]	Antineoplastic	931	Glibenclamide[1234]	Antidiabetic
422	Triaziquone[1234]	Antineoplastic	932	Glibornuride[1234]	Antidiabetic
423	Triethylenemelamine[123]	Antineoplastic	933	Glicaramide[1234]	Antidiabetic
424	Trofosfamide[1234]	Antineoplastic	934	Gliclazide[1234]	Antidiabetic
425	Tucatinib[1234]	Antineoplastic	935	Glimepiride[1234]	Antidiabetic
426	Uramustine[1234]	Antineoplastic	936	Glipizide[1234]	Antidiabetic
427	Valrubicin[12]	Antineoplastic	937	Gliquidone[1234]	Antidiabetic
428	Vandetanib[1234]	Antineoplastic	938	Glisoxepide[1234]	Antidiabetic
429	Vemurafenib[1234]	Antineoplastic	939	Glyclopyramide[1234]	Antidiabetic
430	Venetoclax[1234]	Antineoplastic	940	Glymidine[1234]	Antidiabetic
431	Vinblastine[1234]	Antineoplastic	941	Gosogliptin[1234]	Antidiabetic
432	Vincristine[1234]	Antineoplastic	942	Ipragliflozin[1234]	Antidiabetic
433	Vindesine[1234]	Antineoplastic	943	Linagliptin[1234]	Antidiabetic
434	Vinflunine[1234]	Antineoplastic	944	Lobeglitazone[1234]	Antidiabetic
435	Vinorelbine[1234]	Antineoplastic	945	Luseogliflozin[1234]	Antidiabetic
436	Vismodegib[1234]	Antineoplastic	946	Metahexamide[1234]	Antidiabetic
437	Vorinostat[1234]	Antineoplastic	947	Metformin[1234]	Antidiabetic
438	Vosaroxin[1234]	Antineoplastic	948	Miglitol[1234]	Antidiabetic
439	Zorubicin[12]	Antineoplastic	949	Mitiglinide[1234]	Antidiabetic
440	2,4-Diaminopyrimidine[1]	Antibacterial	950	Muraglitazar[1234]	Antidiabetic
441	Alatrofloxacin[1234]	Antibacterial	951	Nateglinide[1234]	Antidiabetic

442	Amdinocillin[1234]	Antibacterial	952	Netoglitazone[1234]	Antidiabetic
443	Amikacin[1234]	Antibacterial	953	Omarigliptin[1234]	Antidiabetic
444	Amoxicillin[1234]	Antibacterial	954	Phenformin[1234]	Antidiabetic
445	Ampicillin[1234]	Antibacterial	955	Pioglitazone[1234]	Antidiabetic
446	Apramycin[123]	Antibacterial	956	Ranirestat[1234]	Antidiabetic
447	Arbekacin[1234]	Antibacterial	957	Remogliflozin etabonate[1234]	Antidiabetic
448	Aspoxicillin[1234]	Antibacterial	958	Repaglinide[1234]	Antidiabetic
449	Astromicin[1234]	Antibacterial	959	Rivoglitazone[1234]	Antidiabetic
450	Avibactam[1234]	Antibacterial	960	Rosiglitazone[1234]	Antidiabetic
451	Azidamfenicol[1]	Antibacterial	961	Saroglitazar[1234]	Antidiabetic
452	Azidocillin[1234]	Antibacterial	962	Saxagliptin[1234]	Antidiabetic
453	Azithromycin[1234]	Antibacterial	963	Sergliflozin etabonate[1234]	Antidiabetic
454	Azlocillin[1234]	Antibacterial	964	Sitagliptin[1234]	Antidiabetic
455	Aztreonam[1234]	Antibacterial	965	Sotagliflozin[1234]	Antidiabetic
456	Bacampicillin[1234]	Antibacterial	966	Teneligliptin[1234]	Antidiabetic
457	Bacitracin[1234]	Antibacterial	967	Tesaglitazar[1234]	Antidiabetic
458	Balofloxacin[1234]	Antibacterial	968	Thiazolidinedione[123]	Antidiabetic
459	Bekanamycin[1234]	Antibacterial	969	Tofogliflozin[1234]	Antidiabetic
460	Benzylpenicillin[1234]	Antibacterial	970	Tolazamide[1234]	Antidiabetic
461	Besifloxacin[1234]	Antibacterial	971	Tolbutamide[1234]	Antidiabetic
462	Biapenem[1234]	Antibacterial	972	Tolrestat[1234]	Antidiabetic
463	Boromycin[12]	Antibacterial	973	Trelagliptin[1234]	Antidiabetic
464	Brodimoprim[1234]	Antibacterial	974	Troglitazone[1234]	Antidiabetic
465	Carbenicillin[1234]	Antibacterial	975	Vildagliptin[1234]	Antidiabetic
466	Carindacillin[1234]	Antibacterial	976	Voglibose[1234]	Antidiabetic
467	Carumonam[1234]	Antibacterial	977	Zenarestat[123]	Antidiabetic
468	Cefacetrile[1234]	Antibacterial	978	Acebutolol[1234]	Anti-arrhythmic
469	Cefaclor[1234]	Antibacterial	979	Ajmaline[1234]	Anti-arrhythmic
470	Cefadroxil[1234]	Antibacterial	980	Amiodarone[1234]	Anti-arrhythmic
471	Cefalexin	Antibacterial	981	Aprindine[1234]	Anti-arrhythmic
472	Cefaloglycin[1234]	Antibacterial	982	Atenolol[1234]	Anti-arrhythmic
473	Cefalonium[1234]	Antibacterial	983	Atropine[123]	Anti-arrhythmic
474	Cefaloridine[1234]	Antibacterial	984	Bretylium[1234]	Anti-arrhythmic
475	Cefalotin[1234]	Antibacterial	985	Bunaftine[1234]	Anti-arrhythmic
476	Cefamandole[1234]	Antibacterial	986	Celivarone[1234]	Anti-arrhythmic
477	Cefapirin[1234]	Antibacterial	987	Cibenzoline[1234]	Anti-arrhythmic
478	Cefatrizine[1234]	Antibacterial	988	Digitoxin[1234]	Anti-arrhythmic
479	Cefazaflur[1234]	Antibacterial	989	Digoxin[1234]	Anti-arrhythmic
480	Cefazedone[1234]	Antibacterial	990	Diltiazem[1234]	Anti-arrhythmic
481	Cefazolin[1234]	Antibacterial	991	Disopyramide[12]	Anti-arrhythmic
482	Cefbuperazone[1234]	Antibacterial	992	Dofetilide[1234]	Anti-arrhythmic
483	Cefcapene[1234]	Antibacterial	993	Dronedarone[1234]	Anti-arrhythmic
484	Cefdaloxime[1234]	Antibacterial	994	Encainide[1234]	Anti-arrhythmic
485	Cefdinir[1234]	Antibacterial	995	Esmolol[1234]	Anti-arrhythmic
486	Cefditoren[1234]	Antibacterial	996	Ethacizine[1234]	Anti-arrhythmic
487	Cefepime[1234]	Antibacterial	997	Flecainide[1234]	Anti-arrhythmic
488	Cefetamet[1234]	Antibacterial	998	Hydroquinidine[1234]	Anti-arrhythmic
489	Cefixime[1234]	Antibacterial	999	Ibutilide[1234]	Anti-arrhythmic
490	Cefmenoxime[1234]	Antibacterial	1000	Indecainide[12]	Anti-arrhythmic
491	Cefmetazole[1234]	Antibacterial	1001	Lorajmine[1234]	Anti-arrhythmic
492	Cefminox[1234]	Antibacterial	1002	Lorcainide[1234]	Anti-arrhythmic
493	Cefodizime[1234]	Antibacterial	1003	Metoprolol[1234]	Anti-arrhythmic
494	Cefonicid[1234]	Antibacterial	1004	Moracizine[1234]	Anti-arrhythmic
495	Cefoperazone[1234]	Antibacterial	1005	Nadolol[1234]	Anti-arrhythmic
496	Ceforanide[1234]	Antibacterial	1006	Nifekalant[1234]	Anti-arrhythmic
497	Cefotaxime[1234]	Antibacterial	1007	Ouabain[1234]	Anti-arrhythmic
498	Cefotetan[1234]	Antibacterial	1008	Pindolol[1234]	Anti-arrhythmic

499	Cefotiam[1234]	Antibacterial	1009	Prajmaline[1234]	Anti-arrhythmic
500	Cefovecin[123]	Antibacterial	1010	Procainamide[1234]	Anti-arrhythmic
501	Cefoxitin[1234]	Antibacterial	1011	Propafenone[1234]	Anti-arrhythmic
502	Cefozopran[1234]	Antibacterial	1012	Propranolol[12]	Anti-arrhythmic
503	Cefpimizole[1234]	Antibacterial	1013	Quinidine[1234]	Anti-arrhythmic
504	Cefpiramide[1234]	Antibacterial	1014	Sotalol[1234]	Anti-arrhythmic
505	Cefpirome[1234]	Antibacterial	1015	Sparteine[1234]	Anti-arrhythmic
506	Cefpodoxime[1234]	Antibacterial	1016	Tedisamil[1234]	Anti-arrhythmic
507	Cefprozil[1234]	Antibacterial	1017	Tocainide[1234]	Anti-arrhythmic
508	Cefradine[1234]	Antibacterial	1018	Verapamil[1234]	Anti-arrhythmic
509	Cefroxadine[1234]	Antibacterial	1019	Vernakalant[1234]	Anti-arrhythmic
510	Cefsulodin[1234]	Antibacterial			

1. Molecule is in molecular set S_1. **2**. Molecule is in molecular set S_2. **3**. Molecule is in molecular set S_3. **4**. Molecule is in molecular set S_4.

Table S2. Molecules in external validation set.

No.	Name	Category
1	Abiraterone acetate	Antineoplastic
2	Acetylcysteine	Analgesic, antineoplastic, antiviral
3	Anastrozole	Antineoplastic
4	Apalutamide	Antineoplastic
5	Avapritinib	Antineoplastic
6	Berzosertib	Antineoplastic
7	Capmatinib	Antineoplastic
8	Celecoxib	Analgesic, antineoplastic
9	Cyproheptadine	Analgesic
10	Cytarabine	Antineoplastic, antiviral
11	Darolutamide	Antineoplastic
12	Dextromethorphan	Analgesic, antiviral
13	Duvelisib	Antineoplastic
14	Enzalutamide	Antineoplastic
15	Exemestane	Antineoplastic
16	Fulvestrant	Antineoplastic
17	Glasdegib	Antineoplastic
18	Lasmiditan	Analgesic
19	Letrozole	Antineoplastic
20	Lurbinectedin	Antineoplastic
21	Methylergometrine	Analgesic
22	Mobocertinib	Antineoplastic
23	Oliceridine	Analgesic
24	Pomalidomide	Analgesic, antineoplastic
25	Promethazine	Analgesic, antiviral
26	Rifampicin	Antibacterial, antiviral
27	Salicylic acid	Analgesic, antibacterial, antifungal
28	Seliciclib	Antineoplastic, antiviral
29	Sotorasib	Antineoplastic
30	Suxibuzone	Analgesic, antineoplastic
31	Talaporfin	Antineoplastic
32	Tamoxifen	Antineoplastic
33	Tazemetostat	Antineoplastic
34	Tofacitinib	Antineoplastic
35	Ubrogepant	Analgesic
36	Vebicorvir	Antiviral
37	Zanubrutinib	Antineoplastic
